# Local excision followed by early radical surgery in rectal cancer: long-term outcome

**DOI:** 10.1186/s12957-019-1705-6

**Published:** 2019-10-08

**Authors:** Theodor Junginger, Ursula Goenner, Mirjam Hitzler, Tong T. Trinh, Achim Heintz, Daniel Wollschläger

**Affiliations:** 1 Department of General and Abdominal Surgery, University Medical Centre of the Johannes Gutenberg-University, Mainz, Germany; 2 Department of General, Visceral and Vascular Surgery, Catholic Hospital, Mainz, Germany; 3 Department of Heart, Chest and Vascular Surgery, University Medical Centre of the Johannes Gutenberg-University, Mainz, Germany; 4 Institute of Medical Biostatistics, Epidemiology and Informatics, University Medical Centre of the Johannes Gutenberg-University, Langenbeckstr. 1, D 55131 Mainz, Germany

**Keywords:** Rectal cancer, Local excision, Completion surgery, Intraoperative perforation, Oncological outcome

## Abstract

**Background:**

In rectal cancers, radical surgery should follow local excisions, in cases of unexpected, unfavorable tumor characteristics. The oncological results of this completion surgery are inconsistent.

This retrospective cohort study assessed the clinical and long-term oncological outcomes of patients that underwent completion surgery to clarify whether a local excision compromised the results of radical surgery.

**Methods:**

Forty-six patients were included, and the reasons for completion surgery, intraoperative complications, residual tumors, local recurrences (LRs), distant metastases, and cancer-specific survival (CSS) were assessed. The results were compared to 583 patients that underwent primary surgery without adjuvant therapy, treated with a curative intention during the same time period.

**Results:**

The median follow-up was 14.6 years. The reasons for undergoing completion surgery were positive resection margins (24%), high-risk cancer (30%), or both (46%). Intraoperative perforations occurred in 10/46 (22%) cases. Residual tumor in the rectal wall or lymph node involvement occurred in 12/46 (26%) cases. The risk of intraoperative perforation and residual tumor increased with the pT category. Intraoperative perforations did not increase postoperative complications, but they increased the risk of LRs in cases of intramural residual tumors (*p* = 0.003). LRs occurred in 2.6% of pT1/2 and 29% of pT3 tumors. Both the 5- and 10-year CSS rates were 88.8% (95% CI 80.0–98.6). Moreover, the LRs of patients with pT1/2 cancers were lower in patients with completion surgery than in patients with primary surgery.

**Conclusions:**

Rectal wall perforations at the local excision site and residual cancer were the main risks for poor oncological outcomes associated with completion surgery. Local excisions followed by early radical surgery did not appear to compromise outcomes compared to patients with primary surgery for pT1/2 rectal cancer. Improvements in clinical staging should allow more appropriate selection of patients that are eligible for a local excision of rectal cancer.

## Introduction

A local excision is an alternative to radical surgery in patients with low-risk rectal carcinoma [1], and it is increasingly used when patients show good responses to chemoradiotherapy (CRT) [2]. Local excision provides several advantages over radical surgery; for example, it provides lower morbidity and mortality [3]; it carries a lower risk of functional disorders, because it preserves the rectum and anal sphincters [4]; and it avoids an abdomino-perineal resection (APR), which is associated with a permanent colostomy in patients with low rectal cancer.

Increasingly, flexible endoscopy with a submucosal dissection has permitted a surgical-like en bloc resection of superficial rectal lesions [5]. An assessment of the resected specimen can reveal tumor characteristics that determine the risk of lymph node metastases or positive resection margins, which carry a high risk of local recurrence (LR). Early radical surgery can lower the risk of LR [6], and it offers a better prognosis than salvage surgery, in cases of clinically manifest LR [7]. However, evidence on radical surgery strategies and its results has been inconclusive, mainly because we lack large data sets and long follow-up periods. Moreover, there is no consensus about the optimal timing of radical surgery.

A primary problem with this approach is the risk of intraoperative perforation at the local excision site, which might impact the surgical treatment and the outcome. It remains unclear whether an intraoperative perforation raises the risks of postoperative morbidity [8, 9] or the risk of requiring an APR [10, 11]. In addition, it is not clear whether intraoperative perforations compromise oncological outcomes. Furthermore, it is not known whether applying radiotherapy (RT) [11] or CRT [12] before radical surgery might provide any benefit. Randomized studies are lacking.

Therefore, in this study, we performed a retrospective analysis of the results of local excision followed by radical surgery and compared them to the results of primary conventional surgery. This study aimed to (a) investigate the influence of residual cancer and intraoperative perforations at the site of local excision on postoperative clinical and long-term oncological outcomes and (b) clarify whether the oncological results of radical surgery after local excision were inferior to the results of primary conventional surgery for patients treated within the same time period.

## Patients and methods

All patients with rectal cancer were registered in a prospectively maintained database at the Department of General and Abdominal Surgery of the University Medical Center, Mainz (Germany). We identified patients that had received radical completion surgery after a local excision for rectal cancer from 1985 to 2007. Before the local excision was performed, the preoperative diagnosis workup included a digital rectal examination, rigid rectoscopy, endorectal ultrasonography, and abdominal computed tomography. Magnet resonance imaging (MRI) was not routinely performed at the beginning of the study period. The preferred method of local excision was transanal endoscopic full-thickness microsurgery (TEM), with closure of the defect in the rectal wall [13]. Very low tumors were removed with a transanal excision (TAE), performed with a Parks retractor. The indications for a local excision of rectal cancer changed during the study period. When TEM was first introduced in our institution (i.e., the beginning of the TEM era), uT2 tumors were included, in cases of favorable tumor characteristics. Later on, these patients were excluded, according to existing guidelines.

The reasons for early radical surgery were retrospectively classified as follows: (1) a defective local excision (positive margins), which included incomplete resections (R1), unclear margins, or unsafe resection margins (i.e., ≤ 1 mm between the tumor and the deep or lateral resection margin); (2) unfavorable tumor characteristics that indicated high-risk carcinoma, including pT2/3 tumors [14], lymphovascular invasion (L1) [15], perineural invasion, or poor differentiation (G3/4) [16]; or (3) both a defective excision and high-risk carcinoma characteristics.

We collected data on demographic characteristics: the size and location of the tumor in the rectum (locations were based on the distance from the anal verge, as follows: lower third, 0–6.0 cm; middle third, 6.1–12.0 cm; and upper third, 12.1–16 cm); the type of local excision (TEM/TAE, full-thickness/partial resection); the time between the local excision and radical surgery; the reason for radical surgery; intraoperative complications, particularly perforation of the rectal wall; the type of radical surgery (sphincter-saving procedure, low anterior or anterior resection, or APR); postoperative morbidity; and the final pathology assessment [17].

Patients were followed up with regular visits to the oncology department of the hospital, according to a standardized program, until the fifth postoperative year following hospital discharge. Follow-up data were updated for all patients, including those with LR or distant metastasis (DM), in 2012, and 2017. Follow-ups were conducted by contacting the patients, their families, treating physicians, and hospitals. These data included vital status, the presence/absence of disease, the results of follow-up visits, the dates and treatments of tumor recurrences, and the date and cause of death, when applicable.

Local control was determined between the time of radical surgery and a confirmed LR. LR was defined as clinical, radiological, or histological evidence of a recurrent tumor in the local excision site or pelvis, irrespective of DM. DMs were defined as radiological evidence of tumor spread, with or without a LR.

Cancer-specific survival (CSS) was defined as the time from the local excision to death due to rectal cancer. Patients that died from other causes were censored at the time of death. Only tumor recurrence at the time of death and death after surgery due to recurrence were considered events for CSS determinations.

Of 185 patients treated with TEM due to rectal cancer, 42 (22.7%) received completion surgery. Of these 42 patients, 4 were excluded because the radical resection was performed immediately after an intraoperative pathological assessment of the specimen resected with TEM (these 4 patients did not experience LR). Of 28 patients treated with TAE, 8 (28.6%) underwent early radical surgery. Consequently, the present study included 46 patients that received local excisions, followed by early radical surgery, then follow-up examinations, until December 31, 2017. Short-term oncological results of fewer patients were published previously [18].

To compare the oncological outcomes of local excision followed by radical surgery to those of conventional treatment, we identified 583 consecutive patients with pT1-3 rectal cancer that received primary surgery with a curative intention (R0 resection) from 1985 to 2007. These patients did not receive adjuvant therapy. Patients were followed until December 31, 2012.

The total mesorectal excision (TME) technique was introduced in 1996. Thus, both groups included patients with and without TMEs. For comparisons, we reported the results of LRs that occurred after surgeries with and without a TME, in both groups of patients.

### Statistical analysis

Patient characteristics are described as the percentage, median, or mean values. Differences among subgroups of patients with and without LR were assessed with the chi-square test or Fisher’s exact test, as appropriate, for categorical outcomes, and with the Kruskal-Wallis test for continuous outcomes. *p* values were unadjusted for multiple testing. *p* values or confidence intervals (CIs) are presented in the text and tables. The Kaplan–Meier method was used to estimate the probability of CSS over time. Survival times were compared between groups with the log-rank test (univariate analysis). All statistical analyses were performed with the R environment for statistical computing, version 3.5.1 [19].

## Results

Table 1 shows the characteristics of 46 patients that underwent a local excision followed by radical surgery. Table 2 shows the reasons for early completion surgery. TEM was performed in 38/46 (83%) patients. Rectal cancer was located in the middle third of the rectum in most of these patients, but all tumors removed with TAE were located in the lower third of the rectum. In some patients with rectal cancer in the upper third, the rectal wall was partially resected with TEM to avoid opening the peritoneal cavity. In patients with very low rectal cancers, rectal wall was incompletely resected in the region of anal sphincters to avoid injury. The median time interval between the local excision and radical surgery was 21 (range 7–86) days. In 93% (43/46) of patients, radical surgery was performed within 42 days.

**Table 1:** Characteristics of the 46 patients with rectal cancer treated with local excision followed by radical surgery

		Pathological characteristics		
		pT and pN category		
Age (years)				
Median	64.3	pT1	16 (35%)	
Range	33.6–78.0	pT2	23 (50%)	
		pT3	7 (15%)	
Male/female	27/19			
		pN0	37 (80%)	
		pN1	7 (15%)	
Tumor size (cm)		pN2	2 (4%)	
Median	2.8			
Range	0.7–6	pT1N+	1 (6%)	
		pT2N+	4 (17%)	
		pT3N+	4 (57%)	
Tumor site				
Lower third	8 (18%)	Grading		
Middle third	22 (48%)	G1/2	35 (76%)	
Upper third	16 (34%)	G3/4	11 (24%)	
Type of resection		Lymphovascular invasion		
TEM	38 (83%)	L0	4 (9%)	
Full thickness	28	L1	7 (15%)	
Partial	10	LX	35 (76%)	
TAE	8 (17%)			
Full thickness	1			
Partial	6			
Unclear	1			

*TEM* transanal endoscopic micro surgery, *TAE* transanal excision, *LX* lymphovascular invasion not determined

**Table 2:** Causes for early radical surgery following local excision of rectal cancer and residual cancer in resected specimen of 12 patients

Cause of early radical surgery		Residual tumor in rectal wall	Lymph node metastases
*n*	46	6*/46 (13%)	9*/46 (20%)
Positive resection			
Margin**	11 (24%)	0	0
High-risk carcinoma	14 (30%)	0	3 (21%)
Positive resection			
Margin and high-risk carcinoma**	21 (46%)	6* (29%)	6* (29%)
		*p* = 0.02	*p* = 0.055

*3 patients had residual tumor in rectal wall and involved nodes

**Incomplete resection (R1) or indeterminate or unsafe resection margin (minimal distance ≤ 1 mm)

### Radical surgery

All 46 patients received open, non-laparoscopic resections. Of these, 35 (76%) were sphincter-saving procedures, including 2 anterior resections and 33 low anterior resections; of the latter, 8 received hand-sewn coloanal anastomoses. Twenty-two patients received conventional radical surgery without a formal TME, and 24 patients received early TME surgery. In 11 patients (24%), APRs were performed with a permanent colostomy. The percentage of APRs performed depended on tumor location: 59% (10/17) were performed for tumors in the lower third, 4% were performed (1/23) for tumors in the middle third, and none (0/6) were performed for tumors in the upper third. APRs were performed significantly more frequently after a TAE than after a TEM (7/8, 88% vs. 4/38, 11%, *p* < 0.001). Intraoperative perforations of the rectal wall at the local excision sites were observed in 10/46 (22%) patients, and the rate was higher among patients with pT categories (*p* = 0.03; Table 3). The rate of rectal wall perforation was higher with a full-thickness resection (*n* = 8/29, 28%) than with a partial resection (*n* = 1/16, 6%; *p* = 0.1). Intraoperative perforations did not significantly increase the frequency of APRs: APRs were performed in 3/10 (30%) patients with and 8/36 (22%) patients without intraoperative perforations (*p* = 0.7).

**Table 3:** Intraoperative perforation, residual tumor and local recurrence of 46 patients with early radical surgery after local excision of rectal carcinoma

	al	pT1	pT2	pT3	*p*
*n*	46	16	23	7	
Tumor size (cm) (median)	2.8	2.4	3.0	3.0	
Intraoperative perforation	10 (22%)	0	7 (30%)	3 (43%)	0.03
Residual tumor rectal wall	6 (13%)	0	3 (13%)	3 (43%)	0.01
Lymph node metastases	9 (20%)	1 (6%)	4 (17%)	4 (57%)	0.028
Local recurrence	3 (7%)	1 (6%)	0	2 (29%)	0.03

The postoperative course was uneventful in 36 of 46 patients (78%). Ten patients (22%) experienced postoperative complications. There was no postoperative death. Four of the 35 (11%) patients that received sphincter-preserving procedures experienced anastomotic leakage. An intraoperative tear of the rectal wall at the site of local excision had no influence on the rate of postoperative complications (20% with and 22% without intraoperative tears).

### Residual carcinoma

All resected specimens after radical surgery were classified as R0 resections. Seven patients had pT3 tumors (Table 1). Of these, 4 were clinically classified as uT2 and 3 were classified as uT0-1 tumors, based on endorectal sonography before the local excision. In 2 patients (uT0-1), pathological examinations of the local excision revealed a pT1 carcinoma, but perirectal infiltration (pT3) was detected in the resected specimen after radical surgery. Intramural tumors and/or involved lymph nodes were observed in 12/46 (26%) patients.

Among the different reasons for radical surgery, patients with positive resection margins combined with high-risk carcinoma had the highest frequency of residual cancer tissue (9/21, 43%, *p* = 0.008, Table 2). Residual cancer in the rectal wall was detected in 6/46 (13%) patients, and the frequency increased with the degree of tumor infiltration (*p* = 0.01, Table 3). Residual cancer occurred more often after TAE than after TEM (3/8, 38% vs. 3/38, 8%, *p* = 0.06).

In 9 of 46 patients (20%), lymph nodes were involved (N1: *n* = 7, N2: *n* = 2). Similar to the incidence of residual tumors in the rectal wall, the incidence of lymph node metastases increased with the pT category (*p* = 0.028, Table 3). Of the 9 patients with involved nodes, 3 received postoperative adjuvant CRT and 2 received adjuvant chemotherapy.

### Local recurrence

The median follow-up times were 14.7 years for patients that received early conventional surgery and 11.6 years for patients that received early TME surgery. Among patients that survived throughout the study, the median follow-up time was 16.5 years (range 8.6–20.6). LR was observed in 3/46 patients (6.5%), at 0.7, 2.9, and 3.3 years after radical surgery. In all 3 patients, the causes for early radical surgery were incomplete resection (R1) combined with high-risk carcinoma. Two patients with LR had received conventional surgery and 1 had received TME surgery (Table 4). A univariate analysis identified four significant risk factors for LR: the pT category (*p* = 0.03); the pN category (*p* = 0.04); a residual tumor in the rectal wall and involved lymph nodes (*p* = 0.02); and a perforation in the rectal wall, in cases of residual wall tumor (*p* = 0.003). After an intraoperative perforation, LR was observed in both patients with residual tumors in the rectal wall, but not in 8 patients without residual tumors. A multivariate analysis could not be performed, due to the small number of events.

**Table 4:** Local recurrences in patients with primary surgery and patients with local excision followed by radical surgery separated for conventional and TME-surgery (pT1–pT3 cancer of the rectum). The differences within conventional surgery (*p* = 0.2) and TME surgery (*p* = 0.7) were not significant

	Conventional surgery						TME-surgery					
	Primary surgery			LE followed by RS			Primary TME-surgery			LE followed by TME		
	Number	LR (*n*)	Percent	Number	LR (*n*)	Percent	Number	LR (*n*)	Percent	Number	LR (*n*)	Percent
pT1N0	39	1	2.3	11	1	9.1	33	0	0	4	0	0
pT1N+	4	0		0	0		2	0		1	0	
pT2N0	96	11	19.5	7	0	0	97	2	1.9	12	0	0
pT2N+	30	6		2	0		9	0		2	0	
pT1/2 N0	135	12	10.7	18	1	5.0	130	2	1.4	16	0	0
pT1/2 N+	34	6		2	0		11	0		3	0	
pT3N0	103	22	22.6	–	–	50.0	78	10	14.2	3	0	20
pT3N+	56	14		2	1		35	6		2	1	

*LE* Local excision, *TME* Total mesorectal excision, *RS* Radical surgery, *LR* Local recurrence

### Distant metastasis

In 6 of 46 (13%) patients, DMs were observed with (*n* = 1) or without (*n* = 5) LR. The incidence of DMs significantly increased with the pN category (*p* = 0.02), but it was not significantly related to the pT category.

### Survival

**Fig. 1:**
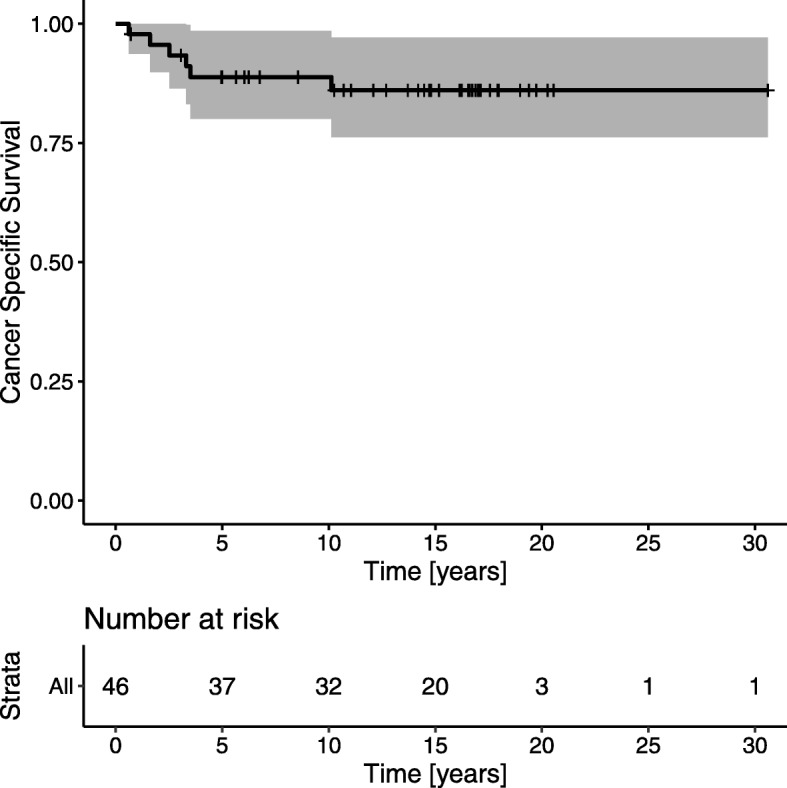
Kaplan–Meier survival analyses show cancer-specific survival for all 46 patients with rectal cancer that received a local excision followed by radical surgery

**Fig. 2:**
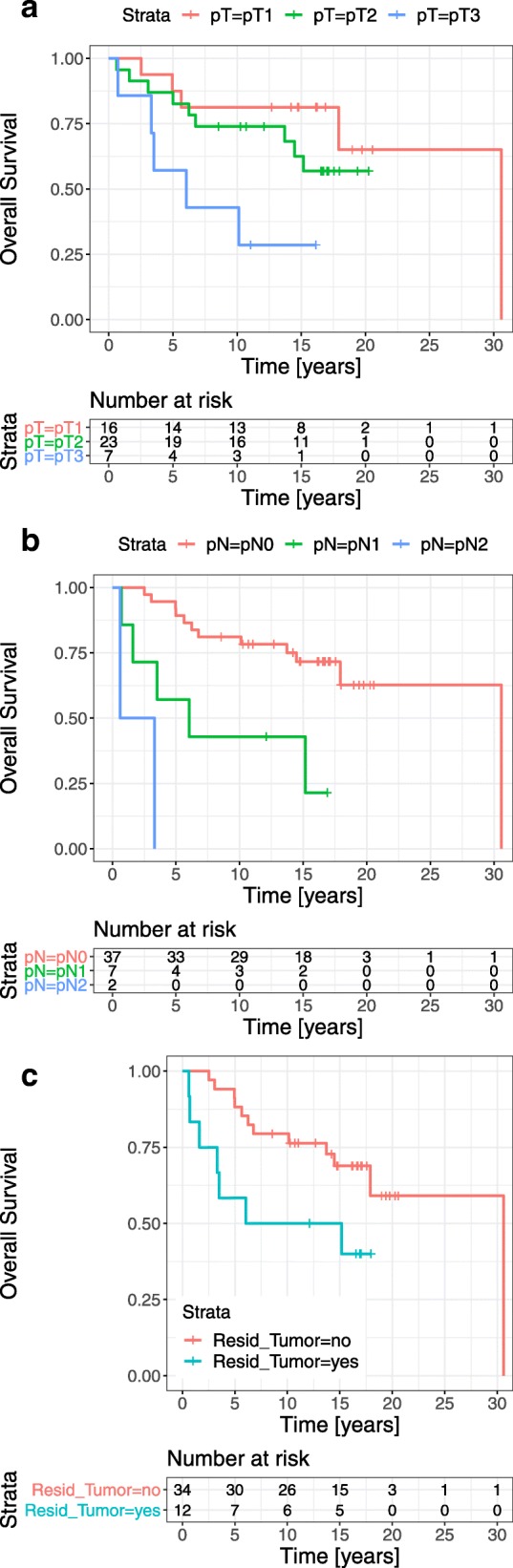
Cancer-specific survival, grouped according to disease category. Survival curves are shown for patients, according to **a** pT categories (*p* = 0.03), **b** pN categories (*p* < 0.001), and **c** the detection of residual intramural tumors and/or lymph node metastases (*p* = 0.008)

At the end of follow-up, 19 of 46 patients (41%) had died. Six deaths were due to rectal cancer, and 13 were due to unrelated diseases. Both the 5- and 10-year CSS rates were 88.0% (95% CI 80.0–98.6; Fig. 1). Both the 5- and 10-year CSS rates were significantly reduced in patients with involved lymph nodes (*p* < 0.001), proven residual tumors (*p* = 0.008), and pT category tumors (*p* = 0.03; Fig. 2a–c).

### Comparison with primary surgery

Out of 583 patients with pT1-3, N0, and N+ rectal cancers that received primary R0 resections, 330 (56.6%) received conventional resections and 253 (43.4%) received TMEs. Inadvertent perforations were observed in 24/583 (4.1%) patients. Sphincter saving procedures were performed in 403/583 (69.1%) patients. The median follow-up was 72 months.

LRs were higher after conventional surgery than after TME surgery. In patients with pT1/2 cancers, LRs occurred less frequently after a local excision followed by surgery than after primary surgery. No LR was observed after LE followed by TME. Conversely, in patients with pT3 cancer, LRs occurred more frequently after local excision followed by surgery than after primary surgery. However, the differences were not significant (Table 4).

## Discussion

Patients with positive margins after local excisions, combined with high-risk carcinoma, had the highest rates of residual cancer at completion surgery. The rates of residual intramural tumor tissue and involved lymph nodes increased with the pT category. An intraoperative opening at the local excision site increased the risk of LR, in cases of residual intramural tumor. The oncological results were in the range of the results of patients that received primary surgery.

There is a general consensus that local excisions, preferably a TEM [20] or transanal minimally invasive microsurgery (TAMIS) [21], are an acceptable alternative to conventional radical surgery for patients with low-risk pT1 rectal carcinoma [1]. Furthermore, it is accepted that radical surgery should follow local excisions, in cases of unexpected unfavorable tumor characteristics [22]. However, radical surgery has some challenging aspects. On one hand, the residual cancer should be eradicated as soon as possible, but, on the other hand, an early second surgical procedure might increase the risk of complications. The rectal wall is weakened after a local excision, and tearing during traction might allow cancer cells to spill out. Moreover, fibrotic scars and adhesions between the mesorectum and the pelvic wall might hinder dissections in the correct plane, and thus, the quality of the resected specimen could be limited [23]. Depending on how these trade-offs were managed, the time between a local excision and radical surgery has varied broadly, from 7 days [24] to 15 weeks [25], in previously reported series. In the present study, early radical surgery was performed within 21 days (median) after a local excision.

A special issue associated with completion surgery after a local excision is the high risk of intraoperative perforation at the local excision site, which may affect the clinical and oncological outcomes. Piessen et al. [8] hypothesized that the practice of leaving the defect unsutured after a TAE contributed to both the high rate of rectal wall tearing (36%) during completion surgery and the high rate of post-surgical complications. They hypothesized that closing the defect would minimize these complications. In the present study, the defect from a TEM was routinely closed after a full-thickness resection. Nevertheless, the rate of intraoperative rectal wall opening was only marginally lower (28%) than that observed by Piessen et al. [8], and it was consistent with the rates observed in other studies on completion surgery [23, 24]. Van Gijn et al. [11] regularly observed perforations during TME following a TEM. A lower perforation rate (6%) might be expected after a partial resection of the muscularis propria, but this approach is not acceptable when a local excision is planned as the only treatment for early rectal carcinoma.

In the present study, intraoperative perforations did not influence the risk of postoperative complications, but they influenced the risk of LR, when residual cancer was present in the rectal wall. It is well known that an intraoperative tumor perforation is a risk factor for LR in patients that undergo primary surgery for rectal cancer [26]. That finding was confirmed in the present study: residual intramural tumors and intraoperative perforations were the most significant risk factors for LR, apart from the pT and pN categories. In patients with pT1 cancer, neither intramural residual tumors nor intraoperative perforations were observed, and the LR rate was in the range of that associated with primary surgery. In patients with pT2 cancer, the risk of perforation increased, but the risk of residual tumor was low, and no LR was observed. Low rates of LR after completion surgery of pT1/2 tumors were also observed in most previous studies [6, 8, 24, 25, 27–29]. Only Gijn et al. [11] reported high LR rates in patients with pT2 cancer. In contrast, patients with pT3 cancer had the highest rates of residual tumors and perforations, which resulted in high LR rates. These rates exceeded the rates observed after primary surgery, even though the difference was not statistically significant. In these patients, endorectal sonography assessments had underestimated the stages. However, over time, as the quality of pre-therapeutic imaging improved, the most advanced rectal cancers should be excluded from the local excision approach. It might also be important to consider the risk of intraoperative perforation for another group of patients: when a local excision is planned after CRT for advanced rectal cancers, patients with poor responses require special care to avoid intraoperative perforations.

Like LR, the rate of DM was associated with the pT and pN categories. Consequently, we found high CSS rates in patients with pT1/2 carcinomas, consistent with former studies [23]. In contrast, patients with pT3 cancers had the highest rates of involved lymph nodes and DM, which significantly lowered the CSS.

All the results of early radical surgery after a local excision were comparable to those of primary surgery, performed during the same time period. This result suggested that a delayed radical surgery after a local excision was not inferior to primary surgery, at least in pT1/2 cancers. Nevertheless, it should be emphasized that the pathological assessments revealed no residual tumors in most patients. To avoid a re-operation after a local excision, a meticulous local excision technique is essential to assure complete resection of all tumor tissues. Additionally, better pre-therapeutic diagnostic methods might clearly reveal the involvement of regional lymph nodes. This improvement is an important future aim that could increase the acceptance of local excision as a curative treatment for rectal cancer.

Our results could not answer the question of whether RT or CRT should be applied after a local excision and before radical surgery. In view of the 2.6% LR rate for all patients with pT1/2 tumors, the benefit of adjuvant therapy might be marginal. In patients with pT3 carcinomas, some results have indicated that a CRT before radical surgery could provide benefit [12], but in current practice, those patients are only seldom selected for local excision surgery.

This study had some limitations. First, it had a retrospective study design, and it was conducted in a single center with a long recruitment time. During that time, TEM was the preferred method of local excision, but TAMIS was introduced as an alternative method. Both methods appeared to provide comparable local excision quality [21]; consequently, the risks of complications after completion surgery that are due to the local excision technique should be comparable for both methods. Moreover, the quality of the TME resection was not determined at the beginning of the TME era. In most patients, no residual tumor was detected in resected specimens; thus, the actual impact of poor mesorectal quality after a completion TME remains unknown [9]. Prognostically, the more important risk factor was an intraoperative perforation in the rectal wall, which was always documented. All patients received open resections, but because laparoscopic and transanal methods (transanal total mesorectal excision) were introduced during the study period, the newer treatments might have conferred more advantages for patients treated later [30]. Finally, the numbers of patients included in the subgroups and the numbers of observed events were small, which limited the conclusions that could be drawn. On the other hand, all consecutive patients were included, and all patients were followed for a very long time period.

Our results suggested that performing completion surgery early after local excisions of pT1/2 rectal cancers with unfavorable tumor characteristics might not compromise the long-term oncological outcome. However, all efforts should be undertaken to avoid re-operations by applying thorough pre-therapeutic diagnostics and performing complete local excisions of early rectal cancers.

## Conclusions

Rectal wall perforations at the local excision site and residual cancer were the main risks for poor oncological outcomes associated with completion surgery. Local excisions followed by early radical surgery did not appear to compromise outcomes compared to patients with primary surgery for pT1/2 rectal cancer. Improvements in clinical staging should allow more appropriate selection of patients that are eligible for a local excision of rectal cancer.

## Data Availability

The datasets used during the current study are available from the corresponding author on reasonable request
